# Impaired belief revision yet intact information seeking in positive schizotypy: A modified task of bias against disconfirmatory evidence

**DOI:** 10.1371/journal.pmen.0000017

**Published:** 2024-09-19

**Authors:** Wanchen Zhao, Wisteria Deng, Tyrone Cannon

**Affiliations:** Department of Psychology, Yale University, New Haven, CT, United States of America; All India Institute of Medical Sciences, INDIA

## Abstract

Cognitive models of delusions emphasize the role of bias against disconfirmatory evidence (BADE) in maintaining false beliefs, but sources of this tendency remain elusive. While impaired information integration could be an explanation for this tendency, the lack of information seeking motive could also result in disregard for new evidence once a (false) belief is formed. The role of information seeking in the association between psychosis-proneness and belief inflexibility has not been investigated in the context of a social interpretation task. In this study, we modified the Interpretation Inflexibility Task (IIT), which assess bias against disconfirmatory evidence in interpersonal contexts, to permit assessment of information seeking by allowing participants to skip seeing increasingly disambiguating information (in the form of pictures at varying degrees of degradation). A robust regression analysis was conducted to examine whether increasing severity of positive schizotypy is associated with more frequent skipping of later trial stages, to examine information seeking. Controlling for the number of pictures seen by participants, a robust mixed effects analysis was conducted to investigate the associations of positive schizotypy, trait anxiety, and the emotional valence of the scenario with a measure of belief revision. Participants higher in positive schizotypy did not opt out of seeing disambiguating information more frequently, *p* = 0.65, *ß* = 0.04; despite this, they still exhibited heightened belief inflexibility by rating the lures and true explanations as equally plausible, *p* < 0.001, *ß* = -0.32. These results suggest that bias against disconfirmatory evidence in positive schizotypy is unlikely a result of reduced information seeking, leaving impaired information integration as a more likely source.

## Introduction

Individuals with schizophrenia and other forms of psychosis show a heightened bias against disconfirmatory evidence (BADE)–defined as a tendency to maintain a false belief in face of information that refutes it [1, 2], reflecting belief inflexibility. What factors contribute to this bias among psychosis-prone individuals? While a general learning deficit may help to explain a heightened reliance on priors in psychosis-like states [3, 4], motivation may be required both to encode new information and fine-tune beliefs. Having reached an initial interpretation of a situation, people with elevated psychosis-proneness (in particular, positive schizotypy) might cognitively disengage from seeking further information. Although this hypothesis has not yet been tested directly, prior work has shown that when given the option to skip trials without penalty on cognitively challenging tasks, individuals with first-episode psychosis skipped more than did healthy controls [5]. Moreover, with a propensity for overconfidence errors [6–8] and strong reliance on prior knowledge [4, 9], those with psychosis-proneness (in particular, positive schizotypy) might be prone to jumping to conclusions about a situation once they form an interpretation based on limited information.

In general, whether an individual engages in information seeking is a function of the instrumental utility, emotional impact, and cognitive relevance; these motivational facets are stable over time and are correlated with general measures of psychopathology [10]. Given that psychosis is associated with motivational deficits [11, 12], bias against disconfirmatory evidence might not only reflect impaired cognitive capacity, but also reduced motivation for information seeking [13–15], because test performance does not solely measure ability [16]. With reduced motivation, individuals prone to psychosis might be less engaged in information presented to them. Therefore, such individuals might not have absorbed all the information on tasks of belief revisioning. Despite extensive research on belief inflexibility in psychosis-proneness, limited work has examined this tendency under the framework of an “information seeking” motive. Conventional paradigms of bias against disconfirmatory evidence present information at several stages, in the format of text or images. Such information is highly ambiguous in the first stage and becomes increasingly disambiguated later. In those paradigms, participants view all stages and rate the plausibility of lures (which are only initially true), true (initially implausible but eventually proven), and absurd explanations at each stage. The plausibility of explanations changes with the disambiguation of information. Despite measuring belief change at each stage, such paradigms are insufficient to study the motivation for information seeking. Thus, current literature in this area cannot untangle the effects of *motivation* versus *capacity* for information processing.

Here we aim to further clarify or rule out information seeking motive as a factor in the association between belief inflexibility and psychosis-proneness. In the current study, the Interpretation Inflexibility Task (IIT) [17] was modified to allow participants to opt out of viewing increasingly disambiguating information. We hypothesized that if a deficit in information seeking underlies bias against disconfirmatory evidence among psychosis-prone individuals, those higher in positive schizotypy would show more trial-skipping in addition to heightened belief inflexibility. We also examined whether bias against disconfirmatory evidence persisted among those higher in positive schizotypy *even when* they chose to view disambiguated information. Trait anxiety increases information seeking behavior [18] and negative affective states associate with lower belief revisioning on scenarios with positive emotional valence [19], despite intact learning with negative valence [20]. In view of these established effects of anxiety on information seeking and belief revision, we include measures of trait anxiety in our study to use as a control variable in later analyses, expecting it to associate with more information seeking and less belief revision on positive interpersonal scenarios.

## Materials and methods

### Participants

The study has been declared exempt from Yale IRB review under 45CFR46.104 (2)(ii), involving minimal risk to participants (IRB protocol ID: 2000026576). All participants gave consent in accordance with Yale IRB guidelines. A sample of 196 participants was recruited from Amazon Mechanical Turk (MTurk), following best practices of online research [21] and sampling from those previously demonstrated good attention according to CloudResearch, including having previously passed sufficient number of attention checks, to prevent bot-like responses and those not following task instructions [22]. After applying exclusion criteria, 175 participants’ data were included in the final analyses (91 female, 83 male, 1 non-binary). Of the excluded participants, 3 were excluded for taking overly long to complete the study (more than 80 minutes); 16 for completing the study within an overly short time frame (less than 30 minutes); and 3 for only using a single rating for plausibility of different explanations on the Interpretation Inflexibility Task. The number of excluded participants was 21 (10% of those who completed the study). Based on a post-hoc power analysis using the R package simr [23], our final sample can achieve at least 80% power for effects of interest, including positive schizotypy, described in data analysis section. **Table 1.** shows participant demographic characteristics. Data for analyses are available at https://github.com/psychack/infoseeking.

**Table 1:** Participant characteristics.

	N (%)
**Gender identity**	
Female	91 (52)
Male	83 (47.43)
Non-binary	1 (0.57)
**Race**	
White	146 (83.43)
Black	19 (10.86)
Asian	5 (2.86)
Multiracial	4 (2.29)
Prefer not to say	1 (0.57)
**Education**	
Less than High School graduate	1 (0.57)
High School or equivalent	13 (7.43)
Some college, no degree	42 (24)
Bachelor’s degree	73 (41.71)
Master’s degree	44 (25.14)
M.D., Ph.D., J.D., or other advanced degrees	2 (1.14)
**Ethnicity**	
Hispanic	17 (9.71)
Not Hispanic	158 (90.29)

The right column shows the number and percent of participants corresponding to each characteristic.

### The Interpretation Inflexibility Task (IIT)

IIT was first introduced by Deng et al. [17] to examine bias against disconfirmatory evidence in paranoia and depression. This task includes 24 sets of pictorially presented interpersonal scenarios (selected from iStock, istockphoto.com), with 12 negative and 12 positive in emotional valence. Each scenario is presented in three stages. In the first stage the picture is presented with 80% blurring, in the second stage with 20% blurring, and in the final stage with no blurring. Participants rated the plausibility of four explanations for the scenario presented in the picture at each stage. Two of the explanations were initially plausible lures, one was absurd, and one was the true explanation. With gradually disambiguated information on each stage, interpretation of the images can change substantially, disproving the lures. **Table 2** shows examples of each type of explanations and links to the corresponding source images. Unlike the original task in which participants viewed all the stages for each scenario, we allowed participants to skip the second or final stages. If participants opted out of seeing the next stage, they completed a filler task and waited for 3 seconds for each stage skipped before moving on.

**Table 2:** Examples of explanations corresponding pictorial scenarios.

	Positive	Negative
Lure 1	People stop you from starting a fight	People laugh at your joke
Lure 2	People are making fun of you	People think you have a great sense of humor
True	People celebrate what a great player you are	People think you can’t tell a joke properly
Absurd	People discuss the smell of the field	The other people give you a comedy award
Source image on iStock	https://www.istockphoto.com/photo/a-soccer-player-celebrates-a-goal-gm1178753201-329578974	https://istockphoto.com/photo/corporate-bullying-business-team-meeting-colleague-gm1132787313-300445499

### Multidimensional Schizotypy Scale—Brief (MSS-B)

MSS-B consists of 38 yes/no questions that measures positive, negative, and disorganized schizotypy. An answer of “yes” yields a score of 1, and “no” yields a score of 0, with reverse coded items adjusted [24]. The average of participants’ answers was calculated for each subscale and for total score. This measure was developed and validated by Gross et al., having demonstrated good internal consistency and exhibited expected patterns of correlations with other clinical measures [24]. In our sample, the MSS-B demonstrated excellent internal consistency, with a Cronbach’s Alpha of 0.93. See **S1–S3 Figs** for distribution of the subscale scores of the study sample.

### State-Trait Anxiety Inventory (STAI)—Trait scale

STAI Trait Scale was developed by Spielberger et al. [25] as part of State-Trait Anxiety Inventory. STAI-Trait Scale consists of 20 statements to measure how anxious people generally feel. Participants responded to those statements on a scale from 1 (almost never) to 4 (almost always). STAI has high internal consistency [26], although it strongly correlates with depressive symptoms and could be considered a general measure of negative affect [27–29]. In our sample STAI exhibits a Cronbach’s Alpha of 0.91. Descriptive statistics for MSS-B and STAI are shown in **Table 3**. See **S4 Fig** for distribution of STAI trait scale scores of the study sample.

**Table 3:** Descriptive statistics for MSS-B and STAI.

	Mean	SD	Range	Median
Negative schizotpy	4.06	3.32	13 (0–13)	4
Positive schizotypy	4.02	4.48	13 (0–13)	3
Disorganized schizotypy	3.26	3.31	12 (0–12)	2
Trait anxiety	44.05	12.77	57 (20–77)	46

### Procedure

Participants entered the study through MTurk and gave informed consent in accordance with the Yale Institutional Review Board guidelines. They then sequentially completed the Interpretation Inflexibility Task, Multidimensional Schizotypy Scale–Brief, and the Trait Scale of State-Trait Anxiety Inventory. At the end of the study, participants were thanked and compensated with $10 for their time.

### Data analysis

#### Belief revision

In our analyses, we used robust regressions and robust mixed effect linear models. To examine the relationship between positive schizotypy and belief revision when allowed to skip trials, we ran a robust mixed effect regression with random intercepts for subjects. We included positive, negative, and disorganized schizotypy, trait anxiety, picture stage (1 to 3, with more information in each stage), condition (positive/negative), total number of pictures seen as independent variables, and rating differences between lures and true explanations as the dependent variable. To compute this dependent variable, we subtracted the average rating of the two lures from the true explanation rating on each trial stage. While a higher difference in rating means higher subjective plausibility of the true explanation over the lures, greater changes in rating differences across picture stages would indicate greater changes in beliefs, as participants become increasingly preferential toward one type of explanation over the other. For example, one might find the lures more plausible in the beginning, leading to a negative rating difference (endorsement of lures over the true explanation) but find the true explanation more plausible in later stages, leading to a positive rating difference.

The current study included schizotypy scores from each MSS-B subscale (positive, negative, and disorganized schizotypy), trait anxiety, stage, condition, number of pictures seen, stage by condition interaction, scores on each MSS-B subscale by stage interaction, trait anxiety by stage interaction, and condition by trait anxiety as independent variables. Because previous studies found extensive evidence for impaired belief revision in paranoia and psychosis-proneness [2, 30–32], subscales were included in the same models to understand how each dimension of psychosis-proneness (suggested by positive schizotypy scores) associate with belief revision. Significant effects of those variables would indicate that participants with schizotypy had different perceptions about the plausibility of true explanations over lures. Stage and condition were included in the model to understand how participants generally update their endorsement of true explanations over lures as information becomes disambiguated and on scenarios with different emotional content. Significant effect of stage would indicate that people increasingly or decreasingly endorsed the true explanation over the lures, suggesting updated beliefs. Trait anxiety was included as a measure of negative affect, because previous research suggested that it increases non-instrumental information seeking during large changes [18] and hampers belief revision on scenarios with positive emotional content [19, 33]. The number of pictures seen by participants were included as measure of information seeking. Higher number of pictures seen would indicate greater motivation for information seeking, having skipped fewer trials. This variable was included to gauge whether information seeking motive affected belief revision.

Stage by condition interactions were included to understand the general tendency for belief revision with different emotional content. The MSS-B subscales (positive, negative, and disorganized schizotypy) by stage interactions reflect whether participants revised their endorsement of the true explanation over lures to a greater extent at later stages. Significant interactions (with a negative t-statistic) would indicate lesser changes in rating differences, implying belief inflexibility. Trait anxiety by stage and trait anxiety by condition interactions were included to understand how it affects interpretation of interpersonal scenarios with different emotional valence; trait anxiety by stage interaction was included to understand whether it is associated with belief inflexibility on both conditions. Given that prior work has found higher bias against disconfirmatory evidence correlates with symptoms of positive schizotypy, such as paranoia, irrespective of emotional valence [30], we did not include the schizotypy by condition interaction. These variables result in the following model (coded in R), using the R package robustlmm [34]:

ratingdifference∼stage+condition+positivesz+negativesz+disorganizedsz+positivesz:stage+negativesz:stage+disorganizedsz:stage+stage:condition+anxiety+anxiety:condition+anxiety:stage+numberofpicturesseen+(1|subject).

To improve power and specifically investigate the effects of psychosis-proneness, we also ran the above model excluding trait anxiety and its related interactions as variables.

To examine moment-to-moment changes in belief only on scenarios which participants voluntarily viewed, we performed a secondary analysis using the metric belief flexibility index [17]. This metric was computed with the following equation on scenarios where participants did not skip any stage [17]. The bias score at each stage was computed by dividing the plausibility rating of the true explanation by the average of ratings of the lures, and then multiplying this value by -1. The bias scores at the three stages will then be used to compute the interpretation flexibility index. Lower value of this index would suggest belief inflexibility. It should be noted that because some participants skipped at least one stage on all scenarios, we have a smaller sample size in this model with 92 participants (N = 83 for negative condition, N = 80 for positive condition).


$$Interpretation\;flexibility\;index=\sqrt{\frac{{\left(bias\;score\;stage\;3-bias\;score\;stage\;2\right)}^{2}+{\left(bias\;score\;stage\;2-bias\;score\;stage\;1\right)}^{2}}{2}}$$

We then ran a robust regression with interpretation flexibility index as the dependent variable, and MSS-B subscale scores (positive, negative, and disorganized schizotypy), trait anxiety, condition, and condition by trait anxiety interaction as the independent variables.

To further investigate positive schizotypy affects belief revisions on different explanation types, we also performed a robust regression with plausibility ratings (raw score) as the dependent variable, in the following model (coded in R):

Rating∼positivesz+explanationtype+explanationtype:stage+positivesz:stage+positivesz:stage:explanationtype

#### Information seeking

Information seeking on the IIT was measured as the number of skipped trials, with higher values reflecting less information seeking behavior. To investigate information seeking motive on the IIT, we ran a robust regression with total skipped trials as dependent variable in the following model (coded in R):

skippedtrials∼positivesz+negativesz+disorganizedsz+anxiety+condition+anxiety:condition

## Results

### Belief revision

**Fig 1:**
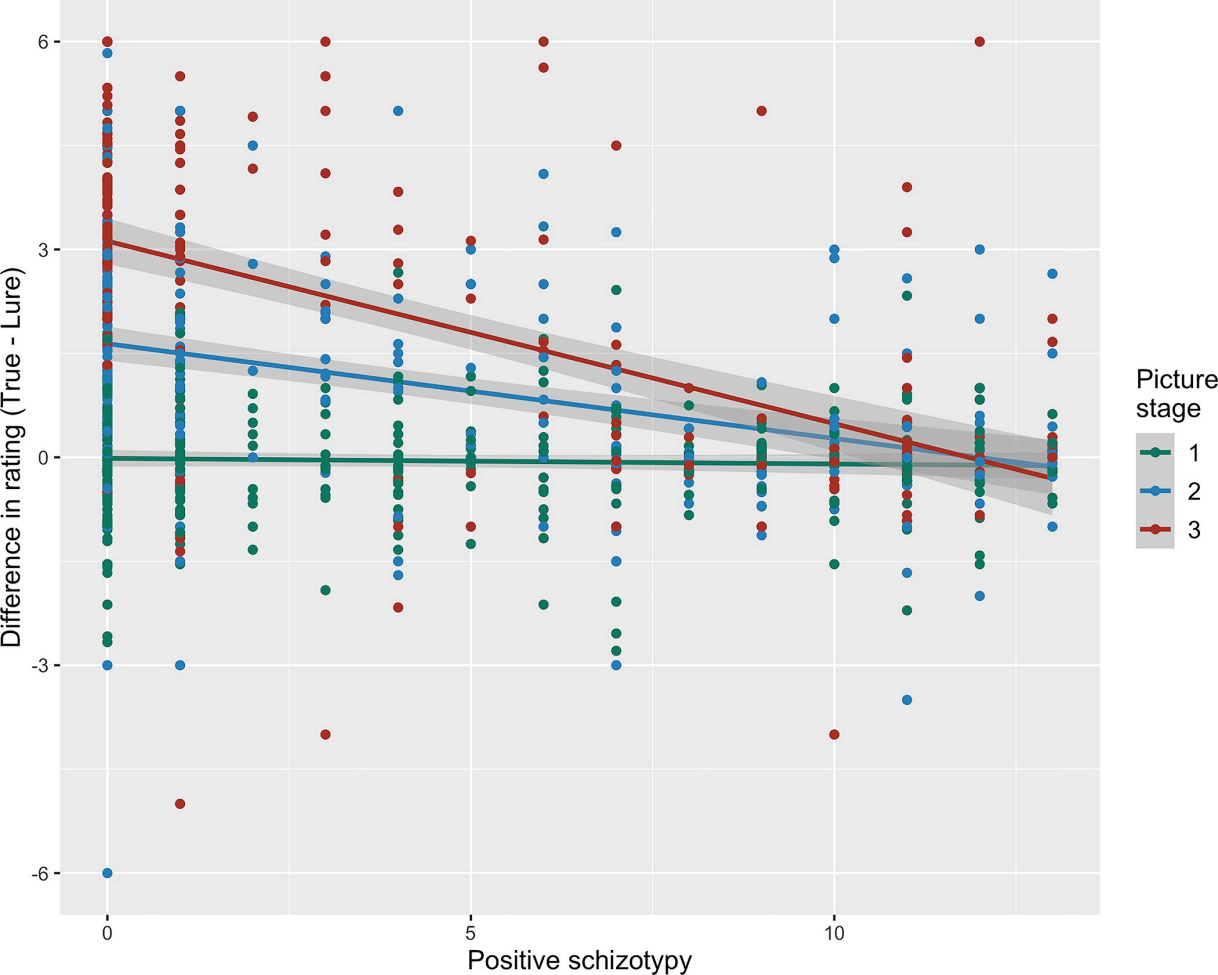
Differences in rating by positive schizotypy and picture stage. Shaded regions indicated 95% confidence intervals. Each dot represents a participant’s response on each stage. Participants generally rate the lures and the true explanation as equally plausible in stage 1, but increasingly shows preference for the true explanation in stage 2 and 3, indicated by rating differences. However, this effect becomes attenuated as positive schizotypy level gets higher. Those with higher positive schizotypy rates the lures and true explanation as equally plausible in all stages.

**Fig 2:**
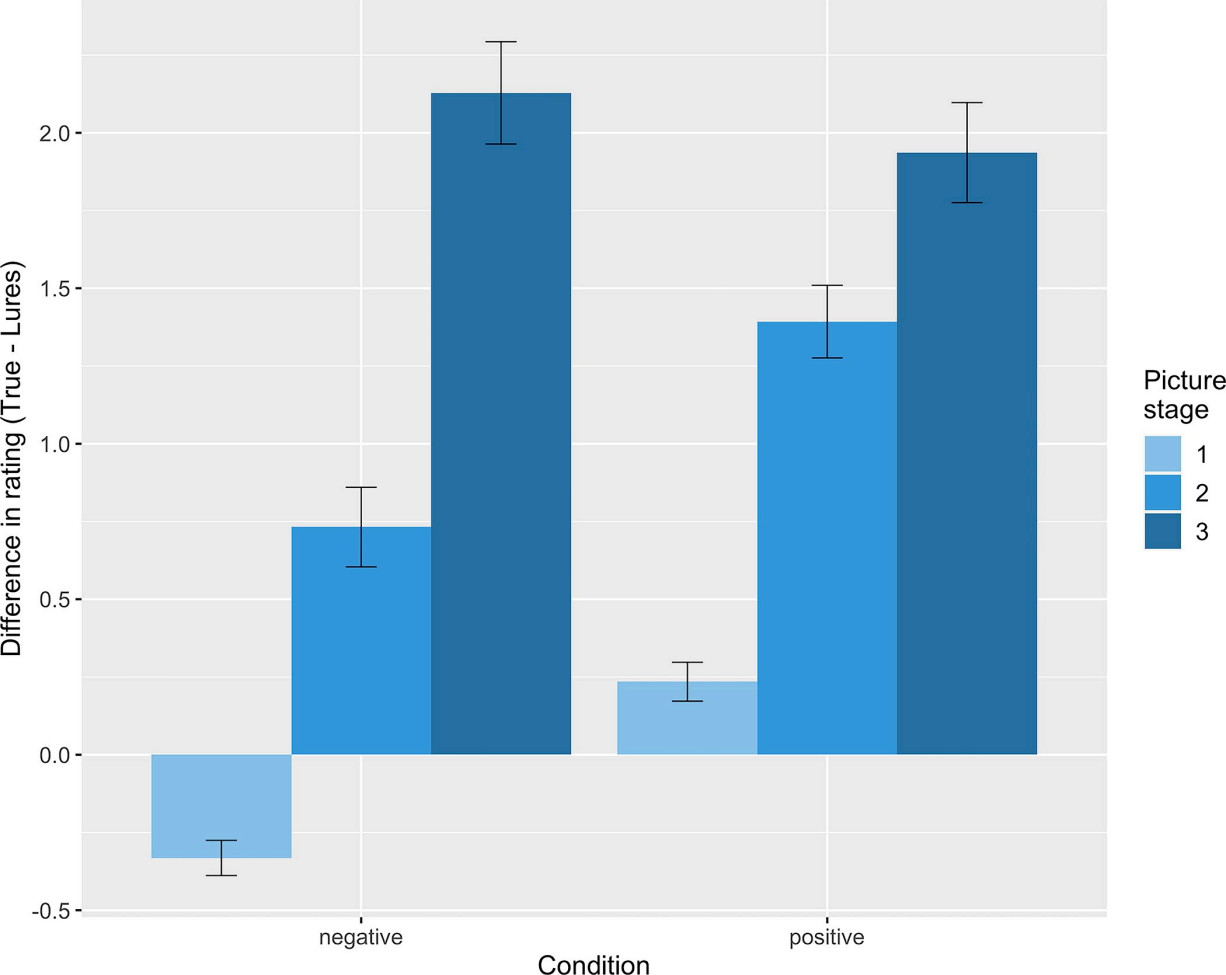
Differences in rating on different picture stages and conditions (negative/positive). Error bars represent one standard error of the mean. Participants shows greater rating differences between the true explanation and lures in stage 2 and 3, suggesting belief revisions. This belief revision occurs earlier in the positive condition, as shown by the greater rating difference increase from stage 1 to stage 2.

We found that participants generally had higher difference ratings (endorsing the true explanation over the lures) for later picture stages, with a large effect size (**Figs 1 and 2**), *t* = 12.24, *p* < 0.001, *ß* = 0.54, 95% CI [0.48, 0.60], suggesting higher endorsement of the true explanation or updated belief in later stages; and for scenarios that resolved to a positive explanation with a small effect size, *t* = 6.70, *p* < 0.001, *ß* = 0.21, 95% CI [0.14, 0.28], such that participants adjust their beliefs more readily for positive emotional content. The picture stage by condition interaction was significant with a small effect size (**Fig 2**), *t* = -3.80, *p* < 0.001, *ß* = -0.14, 95% CI [-0.21, -0.07], such that in positive scenarios, participants preferentially endorsed the true explanation earlier than they did in negative scenarios. See **S1 Table** for descriptive statics on plausibility ratings. See **S5 Fig** for distribution of rating differences.

Although there was a main effect of positive schizotypy on overall rating differences with a medium effect size, *t* = 5.01, *p* < 0.001, *ß* = -0.32, 95% CI [-0.42, -0.23], this was qualified by a significant positive schizotypy by picture stage interaction, *t* = -9.96, *p* < 0.001, *ß* = -0.33, 95% CI [-0.39, -0.26]. As shown in **Fig 1**, participants higher in positive schizotypy continued to rate the lures and true explanations as equally plausible even in later stages of the scenarios, whereas those lower in positive schizotypy gave progressively higher plausibility ratings to true explanations compared with lures across stages. Trait anxiety significantly interacted with condition with a small effect size, such that people with higher trait anxiety had a tendency to endorse the lures over true explanations on scenarios with positive emotional valence, *t* = -4.08, *p* < 0.001, *ß* = -0.15, 95% CI [-0.22, -0.08]. This suggests that trait anxiety associates with less flexible beliefs when scenarios resolve to an emotionally positive explanation.

Although disorganized schizotypy by stage interaction was significant, the effect size was very small, *t* = -2.05, *p* = 0.04, *ß* = -0.06, 95% CI [0.04, 0.17]. In the model where we excluded anxiety, negative schizotypy by stage interaction also became significant with a negligible effect size, *t* = -2.23, *p* = 0.03, *ß* = -0.06, 95% CI [-0.11, -0.01]. The number of pictures seen by participants did not affect rating differences, *p* = 0.53. Detailed robust mixed effects regression results for both models are shown in **Table 4**. See **S2 Table** for regression results with full sample without applying exclusion criteria.

**Table 4:** Robust mixed effects model results on belief revision.

	Full model					Without anxiety				
	Estimate	SE	t	p	Effect size (standardized ß)	Estimate	SE	t	p	Effect size
**Positive schizotypy**	0.16	0.03	5.01	**< 0.001**	-0.32	0.16	0.03	4.95	**< 0.001**	-0.31
Negative schizotypy	0.04	0.03	1.16	0.25	-0.04	0.05	0.03	1.43	0.15	-0.04
**Disorganized schizotypy**	-0.08	0.04	-2.05	**0.04**	0.06	-0.07	0.04	-1.93	**0.05**	0.04
**Stage**	2.04	0.17	12.24	**< 0.001**	0.54	1.81	0.08	21.50	**< 0.001**	0.54
**Condition**	1.81	0.27	6.70	**< 0.001**	0.21	0.89	0.16	5.59	**< 0.001**	0.2
**Trait anxiety**	0.02	0.01	2.07	**0.04**	0.05	-	-	-	-	-
Number of pictures seen	-0.01	0.01	-0.63	0.53	-0.02	-0.01	0.01	-0.56	0.58	-0.01
Stage * condition	-0.30	0.08	-3.80	**< 0.001**	-0.14	-0.30	0.08	-3.68	**< 0.001**	-0.14
Positive SZ * stage	-0.16	0.02	-9.96	**< 0.001**	-0.33	-0.15	0.02	-9.85	**< 0.001**	-0.32
Negative SZ * stage	-0.03	0.02	-1.85	0.07	-0.05	-0.04	0.02	-2.23	**0.03**	-0.06
Disorganized SZ * stage	0.06	0.02	3.15	**0.002**	0.1	0.05	0.02	2.85	**0.004**	0.09
Trait anxiety * condition	-0.02	0.01	-4.08	**< 0.001**	-0.15	-	-	-	**-**	-
Trait anxiety * stage	-0.01	0.00	-1.57	0.12	-0.04	-	-	-	-	-

On scenarios without any skipped stages, participants with higher positive schizotypy had lower belief flexibility index with a large effect size, *t* = -0.15, *p* < 0.001, *ß* = -0.60, 95% CI [-0.80, -0.40], maintaining their previous high plausibility ratings for lures even when contradicted. Other main effects or interactions were not significant, *p* > 0.23. See **S3 Table** for robust regression statistics.

### Plausibility ratings by explanation type

**Fig 3:**
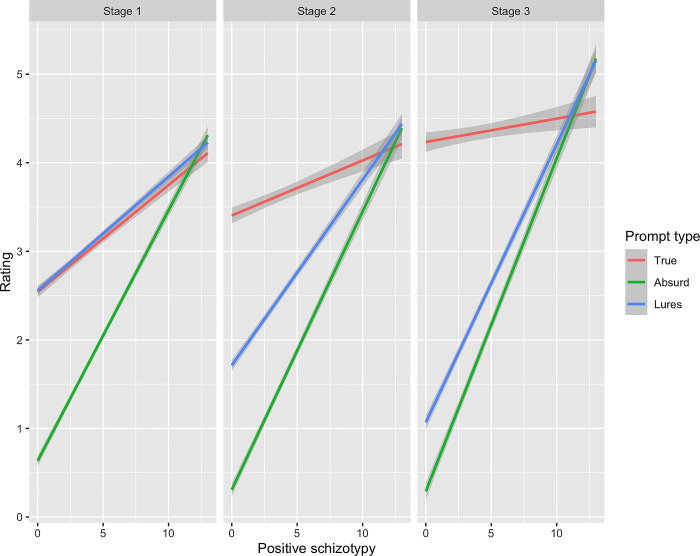
Effect of positive schizotypy on belief revisions across scenario stages and explanation types. Shaded regions represent 95% confidence interval. While participants in general had higher ratings for lures in the first stage and higher ratings for the true explanations in later stages, those with positive schizotypy maintained their high ratings for lures and absurd explanations.

When comparing belief revision on different explanation types, we additionally found participants with higher positive schizotypy generally gave higher plausibility ratings regardless of explanation types or picture stage with a small effect size, *t* = 22.79, *p* < 0.001, *ß* = 0.22, 95% CI [0.20, 0.24], suggesting an indistinct endorsement in all explanations, as shown in **Fig 3**. While participants in general had lower ratings for absurd explanation than the true explanation with a large effect size, *t* = -11.77, *p* < 0.001, *ß* = -0.93, 95% CI [-0.96, -0.91], participants with higher positive schizotypy rated the absurd explanation higher with a large effect size, *t* = 5.63, *p* < 0.001, *ß* = 0.55, 95% CI [0.53, 0.57] (plausibility ratings higher on absurd than true). They also increased their ratings for the explanation to a lesser extent in later stages with a small effect size, *t* = -13.64, *p* < 0.001, *ß* = -0.11, 95% CI [-0.13, -0.10] and maintained their high plausibility ratings for the absurd explanations and lures, *t* = -10.30, *p* < 0.001, *ß* = 0.07, 95% CI [0.06, 0.09], *t* = 31.59, *p* < 0.001, *ß* = 0.18, 95% CI [0.17, 0.19] with a very small effect size, as reflected in the significant three way interactions (positive schizotypy x explanation type x stage) (**Fig 3**). Detailed regression results are shown in **Table 5.** See **S4 Table** for full sample (unexcluded data) statistical model outputs.

**Table 5:** Robust regression on unadjusted plausibility ratings.

	Estimate	SE	t	p	Effect size (ß)
Absurd	-0.85	0.07	-11.77	< 0.001	-0.93
Lure	1.83	0.08	23.66	< 0.001	-0.38
Positive schizotypy	0.20	0.01	22.79	< 0.001	0.22
True*stage	0.99	0.04	28.00	< 0.001	0.29
Absurd*stage	-0.19	0.02	-10.90	< 0.001	-0.01
Lure*stage	-0.86	0.02	-38.33	< 0.001	-0.18
Absurd*positive SZ	0.07	0.01	5.63	< 0.001	0.55
Lure*positive SZ	-0.15	0.01	-14.42	< 0.001	0.27
True*positive SZ*stage	-0.06	0.00	-13.64	< 0.001	-0.11
Absurd*positive SZ*stage	0.04	0.00	10.30	< 0.001	0.07
Lure*positive SZ*stage	0.10	0.00	31.59	< 0.001	0.18

### Information seeking

None of the predictors or their interactions significantly affected the number of trials skipped, *ps* > 0.06. See **S5 Table** for regression results with exclusion criteria applied and **S6 Table** for results without exclusion.

## Discussion

In the current study, we allowed participants to opt out of viewing disambiguating information in an interpersonal belief revision task paradigm. Our goals were to examine information seeking motive in psychosis-proneness and determine whether heightened bias against disconfirmatory evidence persists in those with higher positive schizotypy when information seeking was accounted for. By allowing participants to opt out of seeing disambiguating information, our study was able to parse out the effects of ability and motivation. Our results showed that individuals higher in psychosis-proneness did not avoid information more frequently, but still exhibited more bias against disconfirmatory evidence. Although demonstrating a motivation to obtain information, those higher in positive schizotypy still resisted revising their initial beliefs, by rating the lures and the true explanation as equally plausible even with disambiguated information making the lures less plausible. The indistinct plausibility ratings associated with positive schizotypy might also suggest impaired reasoning capacity or reality testing as cognitive mechanisms for delusion-proneness [35, 36], which involves unusual explanations for events. Mirroring this aspect of impaired reality testing, positive schizotypy associates with persistently high ratings for absurd explanations. With a susceptibility to implausible explanations, people could develop unusual or erroneous thoughts more readily, compatible with delusional symptoms.

Consistent with previous literature, we found belief inflexibility in psychosis-proneness regardless of emotional valence of the scenario, while negative affect (trait anxiety) only contributed to bias against disconfirmatory evidence on positively valenced scenarios. Notably, only positive schizotypy is robustly associated with lower belief updating regardless of emotional valence in interpersonal scenarios, reflected by rating differences across stages and lower belief flexibility index. While negative schizotypy relates more to motivational and cognitive control deficits, positive schizotypy often associates with potent prior expectations and overconfidence that could hamper belief revision and information integration once a false belief is formed. This pattern of belief inflexibility could also be explained by the refusal to downwardly adjust previously established ratings of a scenario [32]. In our sample, participants with higher positive schizotypy were able to increase their ratings for the true explanation but maintained high plausibility ratings for the lures and absurd explanations, consistent with findings of previous research [32]. The present study not only replicates previous findings on belief revision, but also rules out the role of information seeking as an explanation of heightened belief inflexibility in psychosis-proneness.

Different from our initial prediction, we did not find psychosis-proneness to be associated with lower information seeking behavior, at least as operationalized in trial-skipping. Previous studies primarily examined information gathering in psychosis-proneness using the “Beads Task”, where participants prone to psychosis drew fewer beads to reach conclusion about the percentages of beads with a certain color in the jar [37–39]. Such findings suggest lower information seeking motive in psychosis-proneness. However, our results suggest otherwise, using a paradigm which measures both information gathering tendencies and belief revision. While less updated preference towards the true explanation in later stages indicated belief inflexibility, the lack of significance in skipped trials suggested intact information seeking. This inconsistency could have stemmed from differences in task designs. Modifying a paradigm aimed at studying belief (in)flexibility, only three stages were needed for participants to draw a disambiguated conclusion, whereas the Beads Task had much more trials to gather information, allowing more variation in information seeking behavior than our task did. Therefore, the current paradigm could have been less sensitive in detecting differences in information seeking motive, at odds with findings from previous research.

On the other hand, previous studies also suggested that psychosis-proneness reflects risky but not necessarily hasty reasoning, with a tendency to liberally accept all options presented to them [40]. Such liberal acceptance might have explained why participants with higher levels of positive schizotypy exhibited relatively intact information seeking despite their persistently strong endorsement of lures and absurd explanations. Liberally accepting, participants with psychosis-proneness might have perceived all the explanations as plausible, shown by persistent and distinguishably high ratings in the lures and true explanations, leading to the lack rating differences across stages. Additionally, previous studies found overconfidence errors reliably associated with psychosis-proneness [6–8, 35]. Possibly overconfident in their previous plausibility ratings, such participants might also refuse to discount their endorsement of lures and absurd explanations despite disconfirming evidence. This pattern corresponds to a risky (liberally accepting) reasoning, despite not being hasty (intact information seeking).

Given that information seeking, per se, does not appear to account for heightened belief inflexibility in psychosis-prone individuals, processes associated with integrating evidence across multiple stages of disambiguation must play a role. It is possible that belief inflexibility in positive schizotypy reflects a general learning deficit, decreased sensitivity to novel (disconfirmatory) information, or inability to suppress a prior response tendency. Future studies could implement eye-tracking to help isolate attention allocation during each task phase, as people with high positive schizotypy might have solely attended to parts of the pictures that accorded with their initial interpretations.

### Limitations

Several limitations of our study must be acknowledged. Despite following best practices of online research [21], MTurk yields less control over the attention or response quality of participants, potentially weakening the robustness of our findings [41, 42]. Moreover, not having preregistered our study, we did not conduct an a priori power analysis to plan for a sufficiently large sample size. Although a post-hoc power analysis suggests our variables of interest have at least 80% power to detect significance, this approach is less informative of true power [43]. Although it ensures online data quality sampling from those who previously demonstrated good effort for task completion, this method might have filtered out many with higher psychosis-proneness and/or less motivation for information seeking, potentially reducing power to detect an effect related to cognitive effort. Therefore, the association between the level of positive schizotypy and belief inflexibility might not necessarily generalize to those diagnosed with a psychotic disorder.

Because our pictorially presented scenarios only consist of three stages each, the task might not have been cognitively taxing enough for participants to skip viewing, leading to insignificant effects on information seeking. Although we have measured negative affective states with STAI trait scale, we did not measure the effect of depressive symptoms per se, which directly reflects negative emotions and could have influenced belief inflexibility or interacted with symptoms of psychosis. Therefore, our study might not have captured every aspect of belief revision or explored how people with both depressive and psychotic symptoms revise their believes.

Despite those limitations, our study replicated previous findings on belief inflexibility in psychosis-proneness [2, 31], while taking information seeking motive into consideration, confirming impaired information integration as a likely source for belief inflexibility.

## Supporting information

S1 Table Descriptive statistics for IIT plausibility ratings. Positive schizotypy median = 3 on a scale of 0–13. Plausibility ratings range from 0 to 6.
(DOCX)

S2 Table Robust mixed effects model results on belief revision with full sample. (DOCX)

S3 Table Robust regression results on belief flexibility index. (DOCX)

S4 Table Robust regression on unadjusted plausibility ratings with full sample. (DOCX)

S5 Table Linear regression results on skipped trials. Note: N = 175. Statistical output from robust regression on information seeking (measured by number of skipped trials).
(DOCX)

S6 Table Linear regression results on skipped trials with full sample. (DOCX)

S1 Fig Distribution of positive schizotypy. (TIFF)

S2 Fig Distribution of negative schizotypy. (TIFF)

S3 Fig Distribution of disorganized schizotypy. (TIFF)

S4 Fig Distribution of trait anxiety. (TIFF)

S5 Fig Distribution of rating differences between lures and the true explanation on each stage and each condition. (TIFF)
