# Supplementary material for: Impaired belief revision yet intact information seeking in positive schizotypy: A modified task of bias against disconfirmatory evidence
Source: PLOS Ment Health. 2024 Sep 19;1(4):e0000017. doi: 10.1371/journal.pmen.0000017 (PMC12798597; doi:10.1371/journal.pmen.0000017)
Supplement: S1 Table — Positive schizotypy median = 3 on a scale of 0–13. Plausibility ratings range from 0 to 6. (DOCX) [file pmen.0000017.s001.docx]

**S1 Table. Descriptive statistics for IIT plausibility ratings.**

| Rating types | | | Mean (SD) plausibility ratings | |
| --- | --- | --- | --- | --- |
| Condition | Stage | Explanation type | High positive schizotypy (Above median) | Low positive schizotypy (Below median) |
| Negative | Stage 1 | True | 3.22 (1.68) | 2.36 (1.51) |
|  |  | Absurd | 2.87 (1.93) | 0.65 (1.17) |
|  |  | Lures | 3.61 (1.60) | 2.62 (1.66) |
|  | Stage 2 | True | 3.70 (1.67) | 3.28 (1.78) |
|  |  | Absurd | 2.73 (1.97) | 0.33 (0.92) |
|  |  | Lures | 3.43 (1.74) | 1.85 (1.70) |
|  | Stage 3 | True | 4.36 (1.54) | 4.29 (1.75) |
|  |  | Absurd | 3.32 (2.11) | 0.21 (0.82) |
|  |  | Lures | 3.60 (1.96) | 0.98 (1.48) |
| Positive | Stage 1 | True | 3.67 (1.49) | 2.86 (1.57) |
|  |  | Absurd | 2.80 (1.91) | 0.76 (1.20) |
|  |  | Lures | 3.45 (1.61) | 2.61 (1.63) |
|  | Stage 2 | True | 3.97 (1.45) | 3.67 (1.74) |
|  |  | Absurd | 2.81 (1.98) | 0.49 (1.07) |
|  |  | Lures | 3.23 (1.77) | 1.74 (1.65) |
|  | Stage 3 | True | 4.49 (1.35) | 4.24 (1.69) |
|  |  | Absurd | 3.40 (2.10) | 0.54 (1.23) |
|  |  | Lures | 3.68 (1.96) | 1.30 (1.63) |

Positive schizotypy median=3 on a scale of 0 – 13. Plausibility ratings range from 0 to 6.
