# Supplementary material for: Impaired belief revision yet intact information seeking in positive schizotypy: A modified task of bias against disconfirmatory evidence
Source: PLOS Ment Health. 2024 Sep 19;1(4):e0000017. doi: 10.1371/journal.pmen.0000017 (PMC12798597; doi:10.1371/journal.pmen.0000017)
Supplement: S2 Table — (DOCX) [file pmen.0000017.s002.docx]

**S2 Table. Robust mixed effects model results on belief revision with full sample**

|  | Full model | | | | | Without anxiety | | | |  |
| --- | --- | --- | --- | --- | --- | --- | --- | --- | --- | --- |
|  | Estimate | SE | t | p | ß | Estimate | SE | t | p | ß |
| **Positive schizotypy** | 0.15 | 0.03 | 4.62 | **< 0.001** | -0.28 | 0.14 | 0.03 | 4.38 | **< 0.001** | -0.27 |
| Negative schizotypy | 0.05 | 0.03 | 1.63 | 0.103 | -0.06 | 0.07 | 0.03 | 2.04 | 0.04 | -0.07 |
| **Disorganized schizotypy** | -0.07 | 0.04 | -1.94 | 0.053 | 0.06 | -0.06 | 0.04 | -1.55 | 0.12 | 0.03 |
| **Stage** | 2.21 | 0.16 | 14.08 | **< 0.001** | 0.52 | 1.77 | 0.08 | 21.59 | **< 0.001** | 0.51 |
| **Condition** | 1.74 | 0.26 | 6.78 | **< 0.001** | 0.21 | 0.85 | 0.15 | 5.61 | **< 0.001** | 0.2 |
| **Trait anxiety** | 0.02 | 0.01 | 2.92 | **0.004** | 0.02 | - | - | - | - | - |
| Number of pictures seen | 0.00 | 0.01 | 0.06 | 0.949 | 0.001 | 0.00 | 0.01 | 0.10 | 0.92 | 0.003 |
| **Stage * condition** | -0.29 | 0.07 | -3.82 | **< 0.001** | -0.14 | -0.28 | 0.08 | -3.65 | **< 0.001** | -0.13 |
| **Positive SZ * stage** | -0.14 | 0.02 | -9.07 | **< 0.001** | -0.3 | -0.13 | 0.01 | -8.64 | **< 0.001** | -0.28 |
| **Negative SZ * stage** | 0.05 | 0.02 | 3.02 | **0.005** | -0.07 | -0.06 | 0.02 | -3.46 | **0.001** | -0.09 |
| **Disorganized SZ * stage** | -0.05 | 0.02 | -2.85 | **0.003** | 0.1 | 0.04 | 0.02 | 2.23 | **0.03** | 0.07 |
| **Trait anxiety * condition** | 0.05 | 0.02 | 3.02 | **< 0.001** | -0.15 | - | - | - | - | - |
| **Trait anxiety * stage** | -0.01 | 0.00 | -3.18 | **< 0.001** | -0.07 | - | - | - | - | - |

Note: N=19
