# Supplementary material for: Impaired belief revision yet intact information seeking in positive schizotypy: A modified task of bias against disconfirmatory evidence
Source: PLOS Ment Health. 2024 Sep 19;1(4):e0000017. doi: 10.1371/journal.pmen.0000017 (PMC12798597; doi:10.1371/journal.pmen.0000017)
Supplement: S3 Table — (DOCX) [file pmen.0000017.s003.docx]

**S3 Table. Robust regression results on belief flexibility index**

|  | Estimate | SE | t | p | ß |
| --- | --- | --- | --- | --- | --- |
| **Positive schizotypy** | -0.15 | 0.02 | -5.99 | **< 0.001** | -0.6 |
| Negative schizotypy | -0.01 | 0.04 | -0.40 | 0.69 | -0.04 |
| Disorganized schizotypy | 0.00 | 0.03 | -0.05 | 0.96 | -0.01 |
| Condition | 0.62 | 0.54 | 1.14 | 0.26 | 0.23 |
| Trait anxiety | 0.01 | 0.01 | 0.60 | 0.55 | 0.08 |
| Trait anxiety * condition | -0.01 | 0.01 | -0.59 | 0.56 | -0.09 |
| Positive SZ * condition | -0.07 | 0.06 | -1.20 | 0.23 | -0.3 |
| Negative SZ * condition | 0.03 | 0.05 | 0.60 | 0.55 | 0.09 |
| Disorganized SZ * condition | 0.05 | 0.06 | 0.84 | 0.40 | 0.18 |

Note: N = 92.
