# Supplementary material for: Impaired belief revision yet intact information seeking in positive schizotypy: A modified task of bias against disconfirmatory evidence
Source: PLOS Ment Health. 2024 Sep 19;1(4):e0000017. doi: 10.1371/journal.pmen.0000017 (PMC12798597; doi:10.1371/journal.pmen.0000017)
Supplement: S4 Table — (DOCX) [file pmen.0000017.s004.docx]

**S4 Table. Robust regression on unadjusted plausibility ratings with full sample.**

|  | Estimate | SE | t | p | ß |
| --- | --- | --- | --- | --- | --- |
| Absurd | -0.84 | 0.07 | -11.88 | < 0.001 | -0.88 |
| Lure | 1.74 | 0.07 | 23.45 | < 0.001 | -0.35 |
| Positive schizotypy | 0.20 | 0.01 | 23.89 | < 0.001 | 0.23 |
| True*stage | 0.94 | 0.03 | 27.79 | < 0.001 | 0.27 |
| Absurd*stage | -0.20 | 0.02 | -10.42 | < 0.001 | -0.01 |
| Lure*stage | -0.82 | 0.02 | -36.44 | < 0.001 | -0.16 |
| Absurd*positive SZ | 0.07 | 0.01 | 6.30 | < 0.001 | 0.54 |
| Lure*positive SZ | -0.15 | 0.01 | -14.74 | < 0.001 | 0.25 |
| True*positive SZ*stage | -0.06 | 0.00 | -13.80 | < 0.001 | -0.11 |
| Absurd*positive SZ*stage | 0.04 | 0.00 | 11.35 | < 0.001 | 0.07 |
| Lure*positive SZ*stage | 0.10 | 0.00 | 32.12 | < 0.001 | 0.17 |

Note: N=196
