# Supplementary material for: Impaired belief revision yet intact information seeking in positive schizotypy: A modified task of bias against disconfirmatory evidence
Source: PLOS Ment Health. 2024 Sep 19;1(4):e0000017. doi: 10.1371/journal.pmen.0000017 (PMC12798597; doi:10.1371/journal.pmen.0000017)
Supplement: S5 Table — Note: N = 175. Statistical output from robust regression on information seeking (measured by number of skipped trials). (DOCX) [file pmen.0000017.s005.docx]

**S5 Table. Linear regression results on skipped trials**

|  | Estimate | SE | t | p | ß |
| --- | --- | --- | --- | --- | --- |
| Positive schizotypy | 0.09 | 0.19 | 0.45 | 0.65 | 0.04 |
| Negative schizotypy | 0.37 | 0.19 | 1.91 | 0.06 | 0.13 |
| Disorganized schizotypy | 0.06 | 0.24 | 0.27 | 0.79 | 0.03 |
| Trait anxiety | -0.04 | 0.07 | -0.67 | 0.50 | -0.06 |
| Condition | -0.12 | 3.92 | -0.03 | 0.98 | 0.02 |
| Trait anxiety * condition | 0.01 | 0.08 | 0.10 | 0.92 | 0.01 |

Note: N = 175. Statistical output from robust regression on information seeking (measured by number of skipped trials).
