# Supplementary material for: Impaired belief revision yet intact information seeking in positive schizotypy: A modified task of bias against disconfirmatory evidence
Source: PLOS Ment Health. 2024 Sep 19;1(4):e0000017. doi: 10.1371/journal.pmen.0000017 (PMC12798597; doi:10.1371/journal.pmen.0000017)
Supplement: S6 Table — (DOCX) [file pmen.0000017.s006.docx]

**S6 Table. Linear regression results on skipped trials with full sample**

|  | Estimate | SE | t | p | ß |
| --- | --- | --- | --- | --- | --- |
| Positive schizotypy | 0.05 | 0.18 | 0.26 | 0.80 | 0.02 |
| Negative schizotypy | 0.19 | 0.19 | 1.00 | 0.32 | 0.07 |
| Disorganized schizotypy | 0.01 | 0.23 | 0.06 | 0.95 | 0.006 |
| Trait anxiety | -0.04 | 0.06 | -0.68 | 0.50 | -0.05 |
| Condition | -0.23 | 3.63 | -0.06 | 0.95 | 0.03 |
| Trait anxiety * condition | 0.01 | 0.08 | 0.14 | 0.89 | 0.01 |

Note: N=196. Statistical output from robust regression on information seeking (measured by number of skipped trials).
