# Supplementary figures and images for: Impaired belief revision yet intact information seeking in positive schizotypy: A modified task of bias against disconfirmatory evidence
Source: PLOS Ment Health. 2024 Sep 19;1(4):e0000017. doi: 10.1371/journal.pmen.0000017 (PMC12798597; doi:10.1371/journal.pmen.0000017)

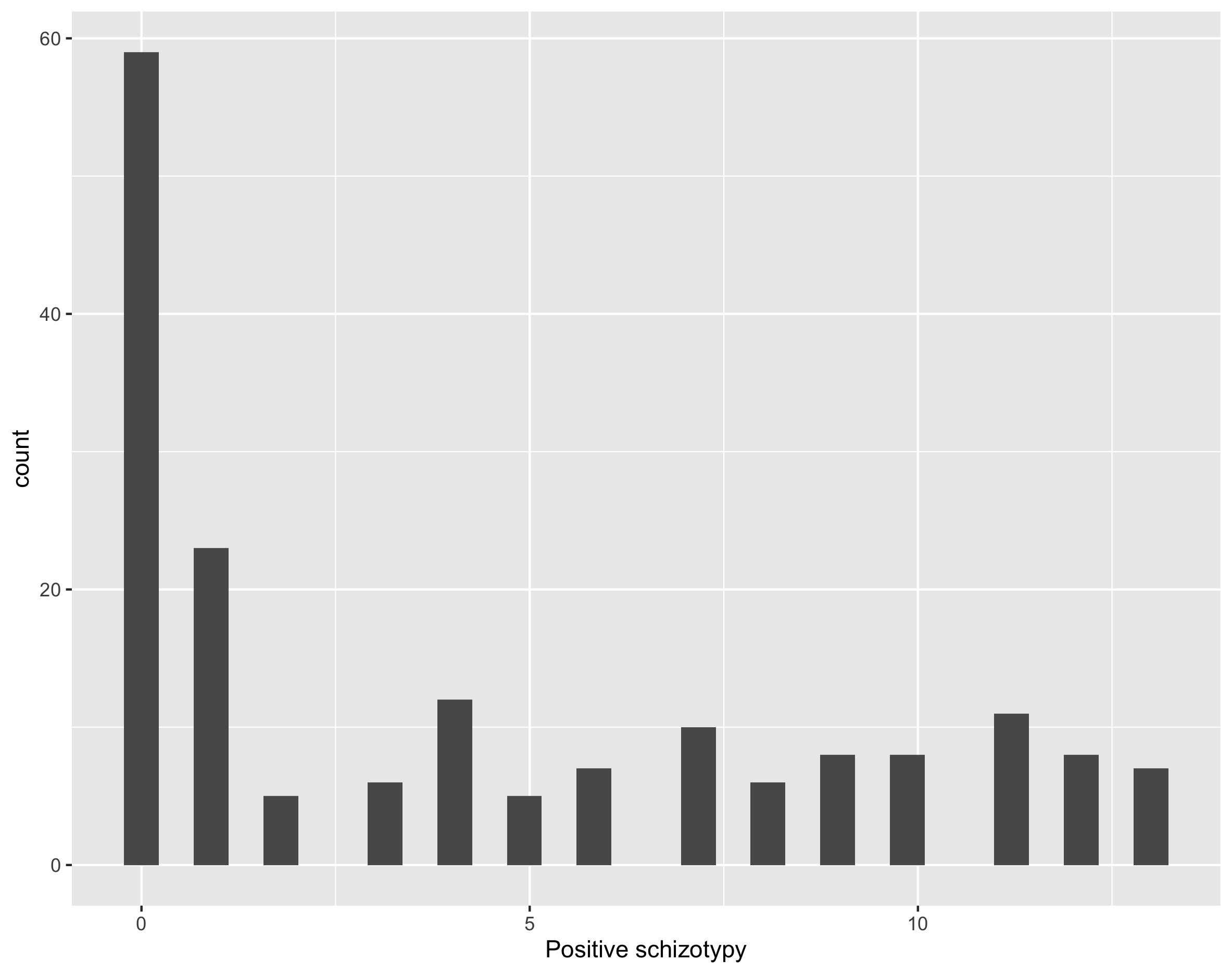

Supplement: S1 Fig — (TIFF) [file pmen.0000017.s007.tiff]

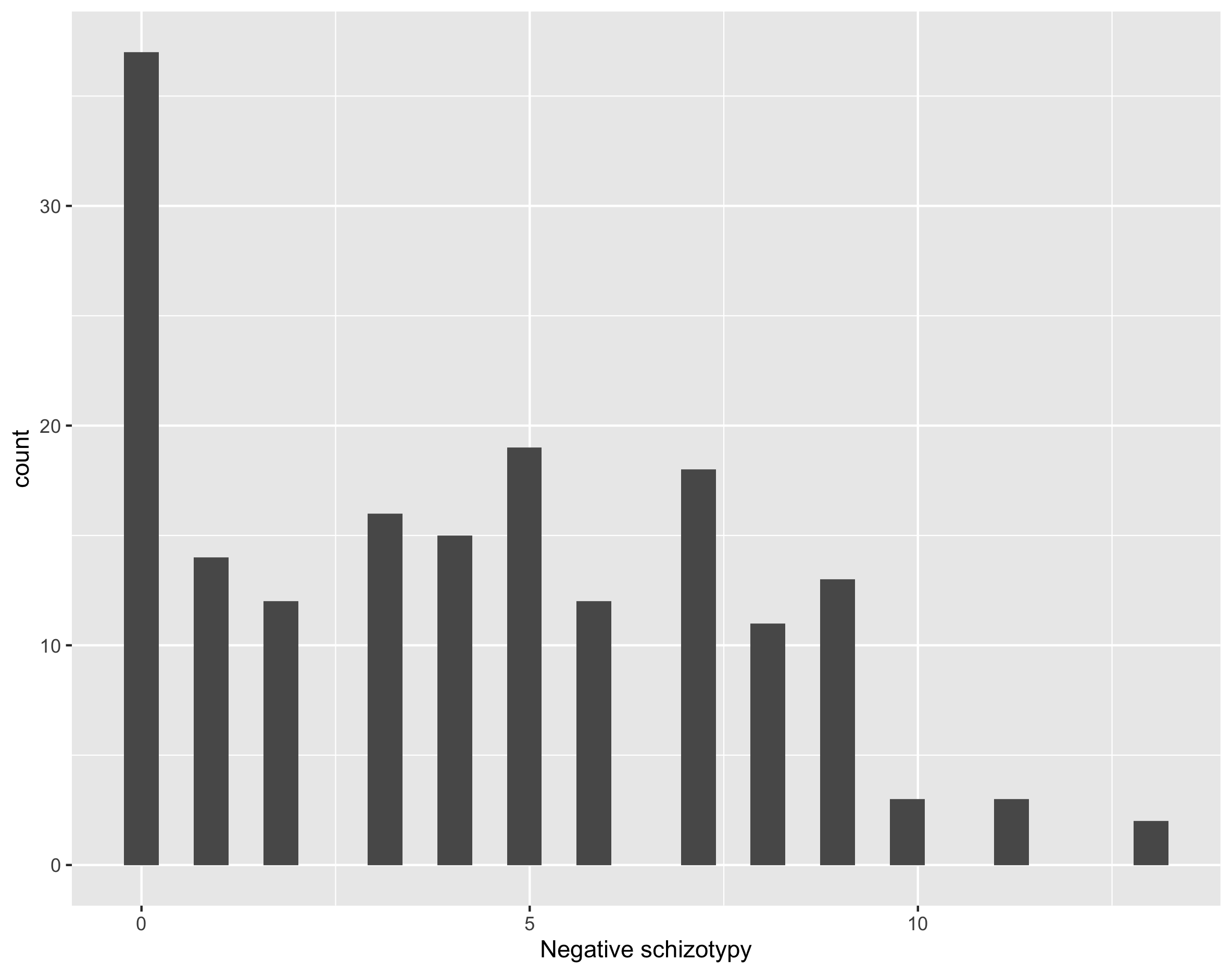

Supplement: S2 Fig — (TIFF) [file pmen.0000017.s008.tiff]

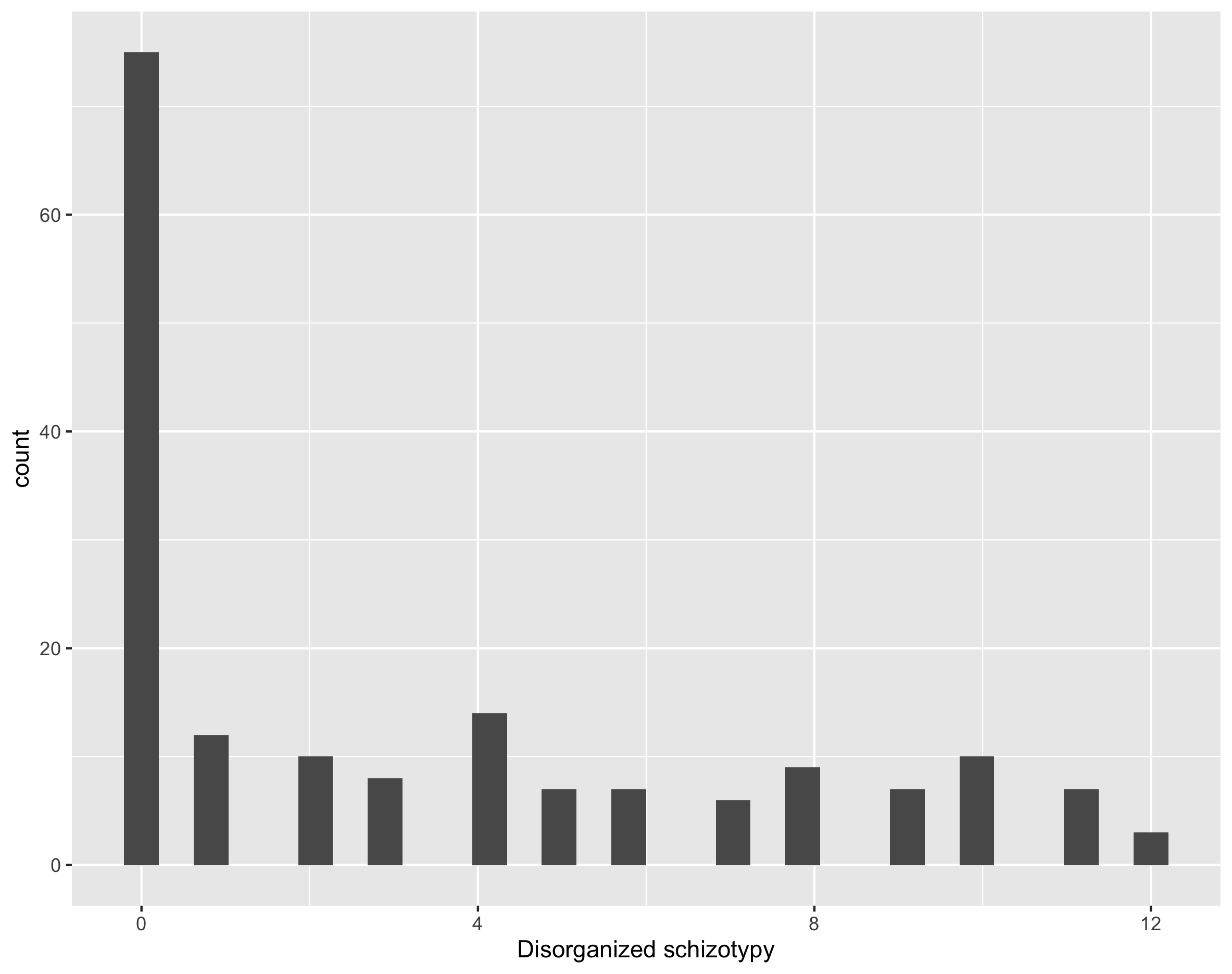

Supplement: S3 Fig — (TIFF) [file pmen.0000017.s009.tiff]

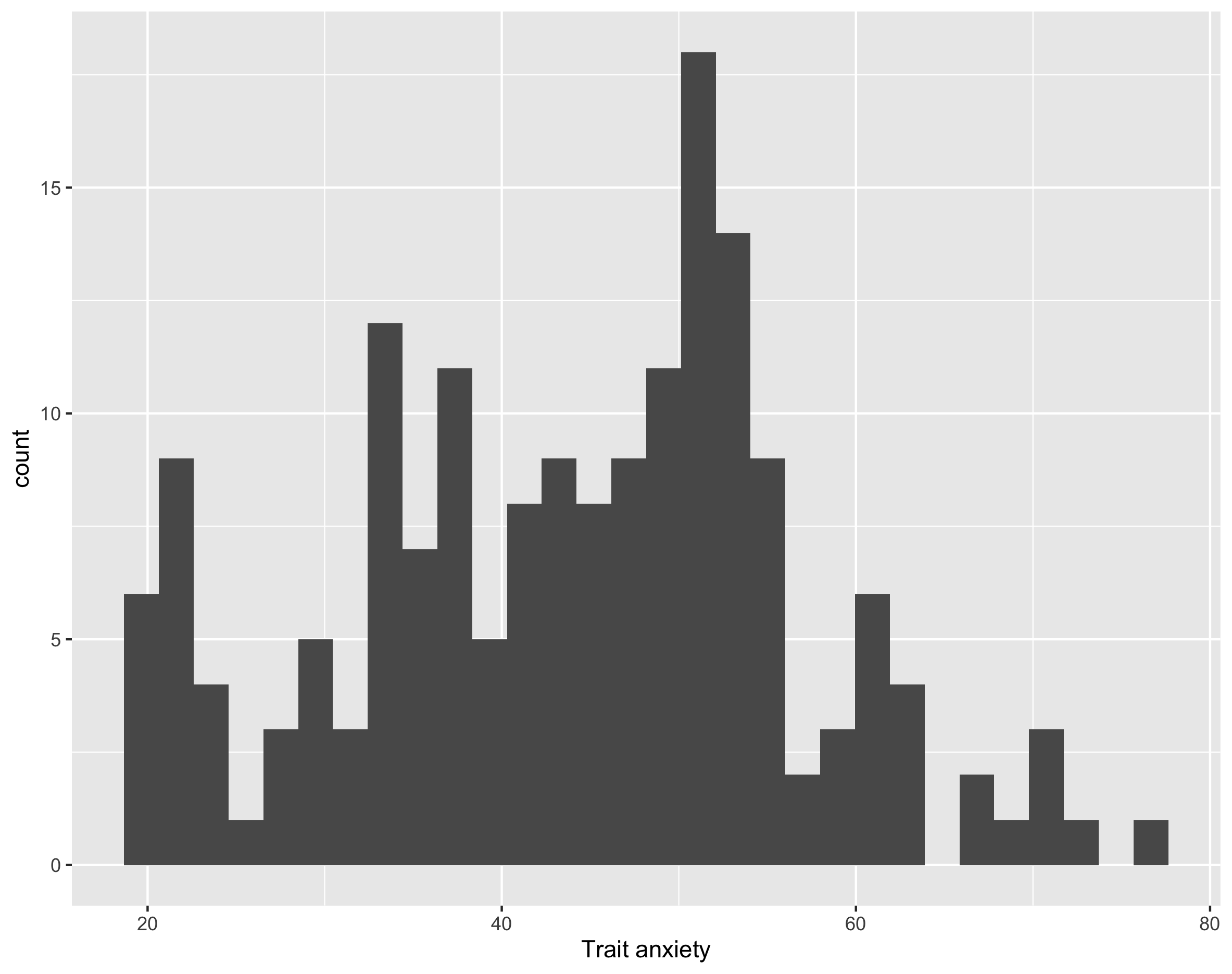

Supplement: S4 Fig — (TIFF) [file pmen.0000017.s010.tiff]

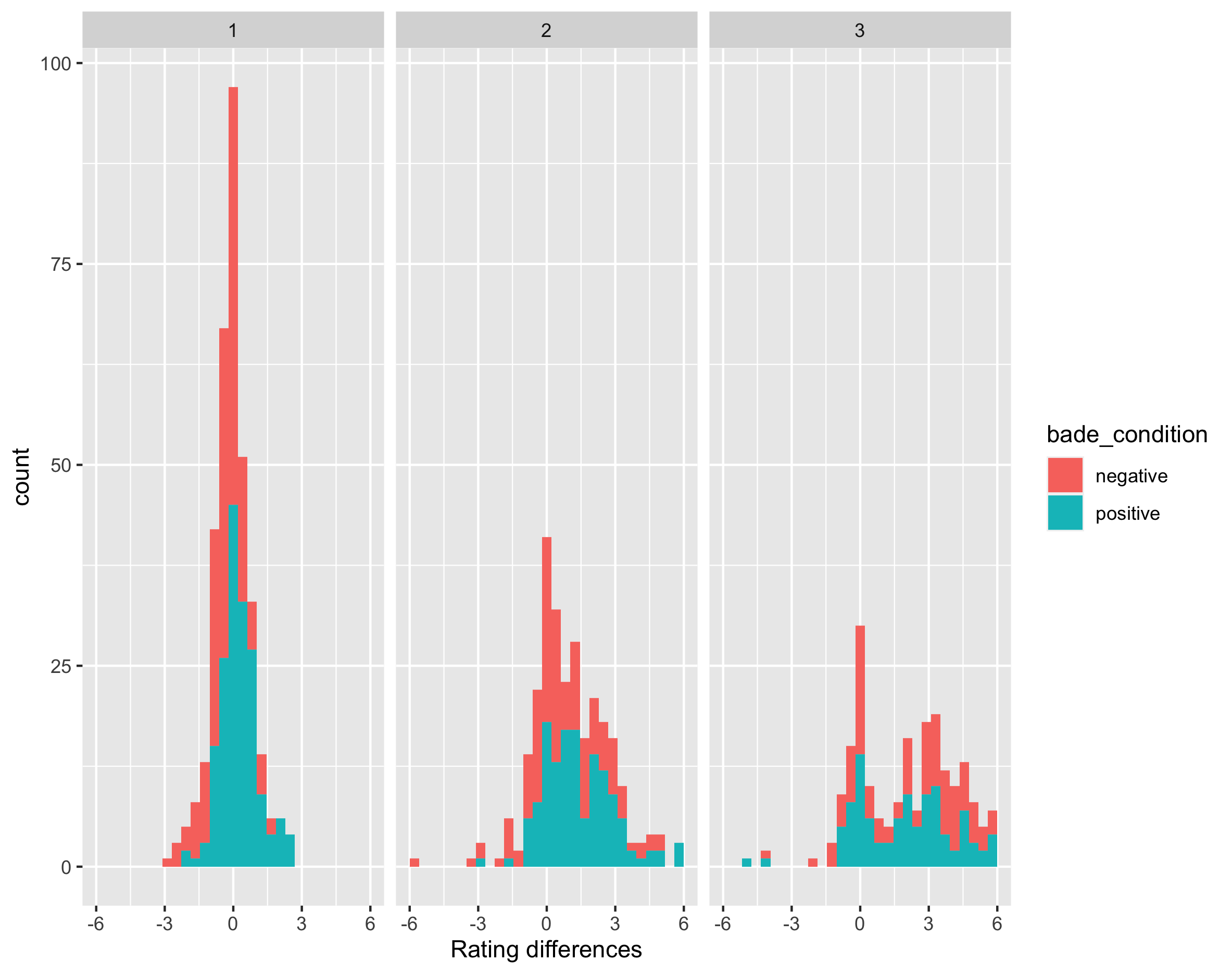

Supplement: S5 Fig — (TIFF) [file pmen.0000017.s011.tiff]
